# Ex-Vivo Preservation of Heart Allografts—An Overview of the Current State

**DOI:** 10.3390/jcdd10030105

**Published:** 2023-03-02

**Authors:** Perin Kothari

**Affiliations:** School of Medicine, Stanford University, Stanford, CA 94305, USA; perin@stanford.edu

**Keywords:** heart transplantation, ex-vivo normothermic perfusion, organ care system, cold ischemic time, hypothermic perfusion

## Abstract

As heart transplantation continues to be the gold-standard therapy for end-stage heart failure, the supply-demand imbalance of available organs worsens. Until recently, there have been no advances in increasing the donor pool, as prolonged cold ischemic time excludes the use of certain donors. The TransMedics Organ Care System (OCS) allows for ex-vivo normothermic perfusion, which allows for a reduction of cold ischemic time and allows for long-distance procurements. Furthermore, the OCS allows for real-time monitoring and assessment of allograft quality, which can be crucial for extended-criteria donors or donation after cardiac death (DCD) donors. Conversely, the XVIVO device allows for hypothermic perfusion to preserve allografts. Despite their limitations, these devices have the potential to alleviate the supply-demand imbalance in donor availability.

## 1. Introduction

End-stage heart failure is the leading cause of mortality among all organ failures worldwide [1]. At present, heart transplantation (HT) remains the gold standard for the treatment of end-stage heart failure, with over 100,000 cases performed since the first successful transplant in 1967 [1]. Due to improvements in immunosuppressive techniques, organ preservation, histocompatibility and matching, and surgical techniques, HT remains a successful and reproducible treatment for end-stage heart failure [2]. After transplantation, the mean survival is now 10–15 years. As a result, as the treatment with the highest survival rate, HT has been growing in the United States and globally, with approximately 3800 and 9000 transplants performed in 2021, respectively. However, as with other solid-organ transplants, a supply-demand imbalance prevents all patients who would benefit from HT from receiving one, and many patients die while on the waiting list. Furthermore, of all consented donors in 2018, only 31% of heart allografts were transplanted [2].

## 2. Current Limitations to Donor Pool

There have been several proposed strategies to increase the donor supply, one of which is using extended-criteria donors. However, these organs have generally been avoided in the past due to unpredictable outcomes. Extended-criteria donors are defined as having hearts with reduced left ventricular ejection fraction from patients with ages greater than 55 years, left ventricular hypertrophy, previous donor cardiac arrest, prolonged ischemic time greater than approximately 4 h, alcohol or substance abuse, and coronary artery disease. Thus, the use of extended-criteria donors may lead to increased risk for recipients [3]. Despite these risks, there is increased pressure to use extended-criteria donors to ease the supply-demand imbalance of available donor hearts.

Heart transplantation for donors after cardiac death (DCD) has also been gaining increased attention. However, these donors have longer warm ischemia periods (the time between withdrawal of life support and the time of administration of the cardioplegia solution), which leads to organ hypoxia, hypoperfusion, and cardiac distension [4]. With these concerns, combined with the inability to assess the graft function prior to implantation, DCD donor hearts have generally been deemed unsuitable for transplantation. However, these hearts would be an ideal platform for ex-vivo perfusion, as it prevents further ischemic injury and allows assessment prior to implantation.

Another limitation for donors is that cold ischemic times are limited to approximately four hours. Cold ischemia is defined as occurring from when the donor heart is arrested until the heart is sewn into the recipient [5]. It has been shown that prolonged cold ischemia times beyond four hours increase the risk of primary graft dysfunction (PGD), and ischemia-reperfusion injury is implicated as one of the main factors leading to PGD [1]. PGD remains the leading cause of 30-day mortality after HT, with prolonged ischemic time being a leading cause of PGD [6]. Thus, organs that are too distant from the recipient location are unsuitable for transplantation.

Nevertheless, cold ischemic storage has become universally adopted since the origins of transplantation nearly 50 years ago. However, it is an imperfect method of storage. Although the cold temperatures decrease the heart’s metabolic requirement, there is some background ATP depletion and the development of lactic acidosis. However, until recently, there has not been an alternative to this simple, reproducible, and inexpensive method of preservation [1].

## 3. Ex-Vivo Normothermic Perfusion—TransMedics Organ Care System

To utilize marginal donors, long-distance procurements, and DCD donors, there is a strategy to avoid prolonged cold ischemic times by using a specialized device that can allow normothermic ex-vivo perfusion during transit from donor to recipient. The only commercially available and approved device to achieve this is the Organ Care System (OCS) made by TransMedics Inc. (Andover, MA, USA). In addition to shortening cold ischemic times, OCS can allow for continuous evaluation of marginal donors, allowing for the exclusion of unsuitable hearts at the recipient site instead of the donor hospital. Another potential advantage of the OCS device is that it gives the surgeon additional time to remove the heart from a re-operative chest or a complex congenital patient that may require additional procedures (e.g. circulatory arrest) to allow for allograft implantation. This extra time, which would normally be added to the cold ischemia time, may now allow surgeons to be more meticulous in dissection, preventing additional bleeding and coagulopathy.

The OCS maintains the donor heart in a warm, contractile, and near-physiologic state during transportation. The harvesting techniques for use of OCS differ slightly between donation after brain death (DBD) and DCD hearts, mostly related to the timing of entering the mediastinum. For DBD donors, sternotomy and pericardiectomy are first completed, after which a visual examination of the biventricular function and palpation of the coronary arteries serve as the final check of allograft quality prior to procurement. Once it is confirmed that the procurement will proceed, the OCS machine is assembled and prepared. Next, a cardioplegia cannula is placed into the aorta, and a venous cannula is placed into the right atrium. Prior to cross-clamp application, approximately 1200–1500 mL of donor blood is drained into a heparinized bag for priming of the OCS circuit. After the left ventricle is vented, the aortic cross clamp is placed, and cardioplegia solution is administered per local institutional protocol. The allograft is then procured in the standard fashion, with incisions at the superior and inferior vena cava, aorta, left atrial cuff, and pulmonary artery.

The protocol for DCD procurement varies slightly. After the injection of 30,000 units of heparin, life support is withdrawn. Per institutional protocol, a certain period is allowed for the patient to enter into electromechanical arrest [2]. If that occurs, then the sternum is entered, and the procedure continues as described above for the DBD donors. However, the time awaiting electromechanical arrest is defined as “warm ischemic time”, as the patient is generally hypotensive and hypoxic to a certain extent.

**Figure 1:**
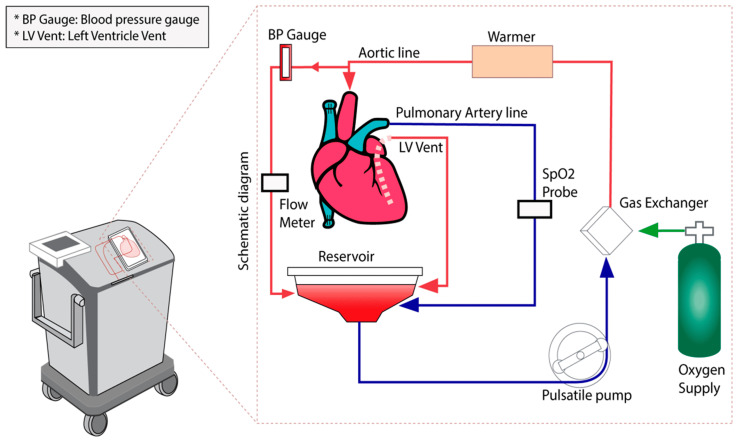
A schematic diagram of the blood flow through the Organ Care System (OCS) system. The blood enters the heart through the aortic cannula and travels retrogradely to the coronary arteries. Deoxygenated blood enters the coronary sinus and drains to the right atrium and right ventricle, where it is ejected out of the pulmonary artery and then drained to the reservoir. There is also a left atrial vent that drains to the reservoir. A pulsatile pump pushes the blood through a gas exchanger and a heater, where the cycle begins again. There is in-line sampling capability from the aortic line.

**Figure 2:**
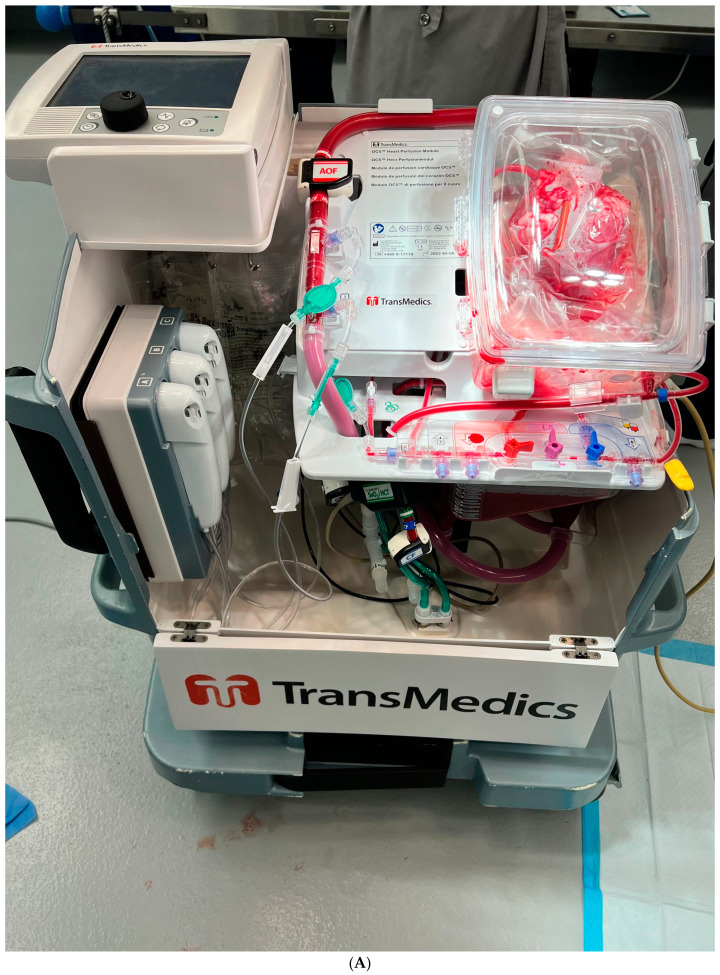

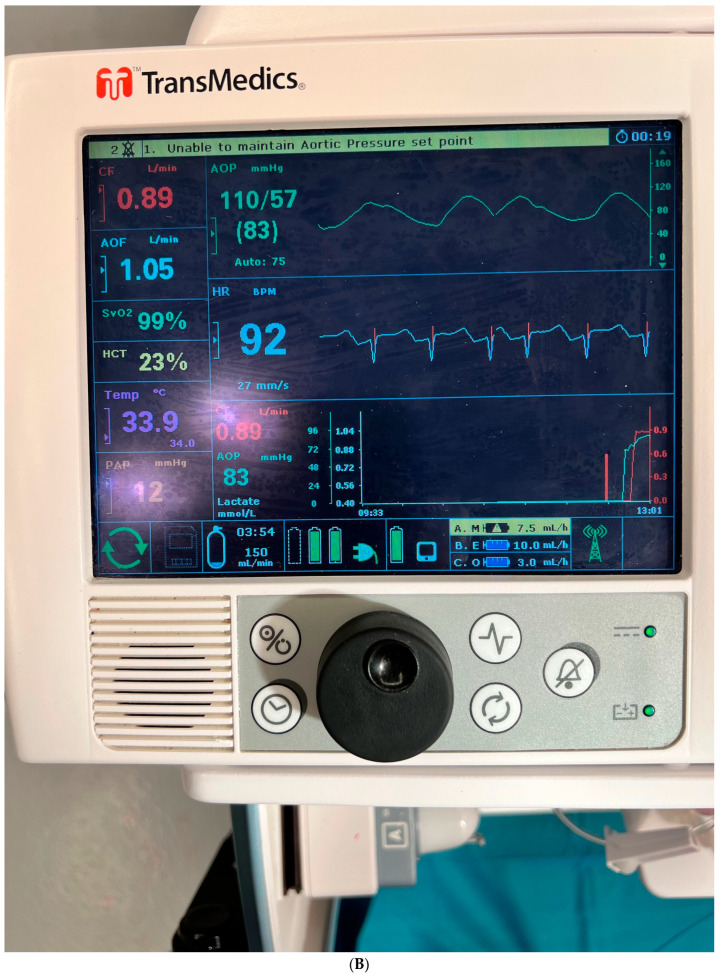
(**A**) Overview of the entire Organ Care System (OCS). Photo courtesy of Brandon Guenthart, MD. (**B**) View of the Organ Care System (OCS) Console. Several parameters are monitored while the allograft is in the console: aortic pressure, coronary flow, heart rate, pump flow, hematocrit, mixed venous oxygen saturation, blood temperature, and lactate levels. Photo courtesy of Brandon Guenthart, MD.

After the heart has been removed from the donor, it is moved to a back table where it is prepared for attachment to the OCS system. The superior and inferior vena cava are sutured closed, the aorta and pulmonary arteries are cannulated, and the heart is placed on the OCS machine with the left ventricle facing upwards to best visualize function. The left ventricle is vented through the left atrium and mitral valve. The aortic and pulmonary cannulas are attached to the circuit, and coronary perfusion with oxygenated blood begins (Figure 1). This blood is mixed with a proprietary solution that contains electrolytes, dextrose, heparin, steroids, insulin, and antibiotics to maintain the organ. Generally, after a few minutes of perfusion, the heart begins contracting. However, there are built-in defibrillation pads in the OCS that can allow for conversion to sinus rhythm with 5 J defibrillation shocks that can be increased in 5 J increments [7]. Ventricular pacing wires are then placed to allow for pacing at a suitable heart rate. As the coronaries begin to be perfused, the venous return drains to the right atrium via the coronary sinus. The blood travels through the tricuspid valve, is pumped out of the right ventricle, and exits the heart through the pulmonary arterial cannula. This allows the coronary perfusion to behave as a closed circuit, allowing for coronary blood flow titration. The ideal coronary blood flow rate is approximately 700–800 mL/min (Figure 2A) [8].

The allograft can be assessed by several methods summarized in Table 1. There is a module on the OCS device that displays real-time measurements such as aortic pressure, coronary flow, blood temperature, and heart rate [3]. Laboratory methods that are used include measuring lactate and lactate differential between arterial and venous blood, as well as potassium and calcium levels (Figure 2B). Donors with unknown coronary artery disease can undergo ex vivo angiography with C-arm fluoroscopy [9]. In addition, at the recipient site, the surgeon can evaluate the clinical function of the allograft prior to implantation. Once the allograft is deemed adequate to proceed, the OCS blood flow is reduced, and cardioplegia solution is administered through the aortic cannula to arrest the heart. The OCS cannula is removed, and the heart is prepared for implantation.

## 4. Literature Review of the OCS Device and Its Outcomes

At present, the literature supports the use of OCS. By 2021, there will have been approximately 1100 transplanted hearts using the OCS, of which 800 were from DBD donors and 300 were from DCD donors [9]. The PROTECT II trial was a prospective multi-institutional study that compared standard-criteria hearts implanted with cold storage vs. OCS. The 2-year survival was 89% in the OCS group and 79% in the control group. The PGD incidence was similar in both groups [10]. The PROCEED II Trial was a prospective multi-institutional noninferiority trial that compared OCS to cold storage. The 30-day patient survival was 94% in the OCS group and 97% in the control group. The short-term clinical outcomes demonstrated that OCS was noninferior to cold storage. A follow-up study from PROCEED II demonstrated no difference in two-year survival or major adverse cardiac events between the two groups [11].

In extended criteria donors, use of OCS demonstrated a significant advantage over static cold storage allografts. The EXPAND-Heart Trial demonstrated that extended-criteria hearts that utilize OCS had a 94% 30-day survival rate and an 88% 6-month survival rate. The incidence of severe primary graft dysfunction was 11% [12].

Langmurr et al. recently published a meta-analysis of all available OCS data. Overall, the data demonstrated that there was no difference in early or late survival in patients transplanted with DCD + OCS vs. DBD + OCS heart allografts vs. static cold storage. One-year survival for the DBD + OCS group was 84% and 89% in the DCD + OCS group, as compared to 87% in the static cold storage cohort. The pooled 4-year survival did not vary significantly between groups either. Secondary outcomes, such as ICU length of stay, hospital length of stay, and primary graft dysfunction, were not statistically significant between the groups [13].

## 5. Limitations Ex-Vivo Normothermic Perfusion

However, there are some practical considerations and limitations that need to be discussed before OCS can become a more routinely utilized resource. At present, the cost of heart transplantation can exceed 1.5 million dollars. The OCS would add upwards of $60,000–$80,000 to that total [14]. Notably, in animal models, increased time on the OCS device demonstrated declining graft functions. However, this has not been clinically correlated in human studies [11]. Furthermore, the OCS is labor-intensive and requires constant active evaluation of the donor heart by checking lab samples and adjusting flow rates by specially trained personnel. Any technical or mechanical errors can result in increased warm ischemic time [15].

Recent studies have also questioned the effectiveness of lactate levels in predicting outcomes after OCS use. Cernic et al. retrospectively examined recipients of DCD organs and their dependency upon mechanical circulatory support (MCS) after implantation. They found that there was no difference in lactate profiles between the two groups. Though OCS uses lactate concentrations as a predictor of allograft quality, this study emphasizes that those values are not predictors of MCS requirements post-transplant. Nevertheless, the conclusion found by Hamed et al. in 2009 remains valid because that study found that lactate values >4.96 mmol/L were the most reliable predictors of 30-day graft failure in DBD heart transplantations [16].

Another study examined PGD incidence and significance in all heart transplants over three years across six centers in the United Kingdom. The authors noted that, while the use of OCS did not increase the overall incidence of PGD, allografts that spent longer times attached to OCS were associated with an increase in PGD [17]. Finally, during transport, there is a requirement for a steady power supply to ensure operation of the OCS, which can complicate transport needs. When considering capital and resource allocation, smaller volume centers may be reluctant to utilize OCS. Furthermore, although the donor heart can be evaluated by the transplant surgeon at the recipient site, the heart is being perfused in a retrograde fashion and is in an unloaded state. Thus, visual contractility may not necessarily be a reliable indicator of how the allograft will function after implantation [15].

## 6. Ex-Vivo Hypothermic Perfusion—XVIVO Device

While ex-vivo normothermic preservation systems are the focus of this article, the ex-vivo hypothermic perfusion system must be discussed. This system was originally created by scientists from Skane University Hospital in Lund, Sweden. Since then, XVIVO Perfusion (Goteborg, Sweden) has purchased the rights to the device. At present, the device is not available for use in the United States. Briefly, the XVIVO system continuously perfuses the heart with a cold, oxygenated cardioplegic solution that includes nutrients, hormones, and red blood cells to maintain a hematocrit of 15%. The device is designed to maintain a mean aortic root pressure of 20 mmHg and allow for coronary blood flow between 150 and 200 mL/min in a non-beating, static state. This device enables continuous perfusion with oxygenated flow while also reducing the metabolic requirements of hypothermic controlled-temperature storage. 

There are promising results that are reported by Nilsson et al. from the first in-human use of the device. The authors found all recipients survived without major adverse events in the first six months and no difference in early graft dysfunction as compared to SCS. However, the XVIVO group had an increased incidence of postoperative kidney dysfunction. The trial had several limitations. For example, it was nonrandomized, thus there is a risk of selection bias for both donors and recipients [18].

A major concern of this technique is edema formation due to elevated perfusion pressure and the oncotic pressure difference of the perfusate. Unfortunately, this edema is interstitial in nature and is less likely to be reversible [15]. Nevertheless, the XVIVO device is less reliant on the technical expertise of the user and negates the need for continuous monitoring and a power source during transport. Future studies must compare this device to the OCS and SCS to establish itself as a viable alternative. In addition, it also must be tested on extended-criteria donors as well as DCD donors.

## 7. Future Directions

The supply-demand imbalance of heart allografts available for transplantation grows as more patients are listed and surgical and perioperative care improves outcomes. However, there has been little progress in increasing the supply of these organs until recently. Ex-vivo normothermic perfusion devices, such as TransMedics OCS, allow for reduced cold ischemic time, long-distance procurements or complex dissections of the recipient, real-time monitoring of allograft quality, and evaluation of extended-criteria donors [19]. It can also be used to further evaluate organs from DCD donors, which normally carry a higher risk due to their long, warm ischemic times. The OCS will allow for continual reassessment of these organs. Normothermic ex-vivo heart perfusion that keeps the heart in a near physiologic state can be a significant breakthrough to expand the donor heart allograft pool and potentially ameliorate the supply-demand mismatch for heart allografts, even though it carries some limitations. Although hypothermic perfusion devices are being introduced, their outcome comparability must be established. Nevertheless, ex-vivo perfusion devices have long-term implications and have a bright future for thoracic transplantation, allowing for more favorable organ utilization and potentially expanding the donor pool.

## Figures and Tables

**Table 1:** Allograft assessment techniques.

OCS Module Measurements	Laboratory Studies	Surgical Interrogation
Aortic pressure	Lactate absolute value	Ex-vivo coronary angiography
Coronary flow	Lactate differential between arterial and venous blood	Clinical evaluation
Blood temperature	Calcium levels	
Heart rate	Potassium levels	
Mixed venous oxygen saturation		
Pulmonary artery pressures		

## Data Availability

Not applicable.
